# Expanding a Behavioral View on Digital Health Access: Drivers and Strategies to Promote Equity

**DOI:** 10.2196/51355

**Published:** 2024-08-01

**Authors:** Maura M Kepper, Lauren A Fowler, Isabelle S Kusters, Jean W Davis, Manal Baqer, Sara Sagui-Henson, Yunyu Xiao, Adati Tarfa, Jean C Yi, Bryan Gibson, Kristin E Heron, Nicole M Alberts, Marissa Burgermaster, Veronica PS Njie-Carr, Lisa M Klesges

**Affiliations:** 1 Prevention Research Center Washington University in St. Louis St. Louis, MO United States; 2 Sexuality, Health, and Gender Center Washington University in St. Louis School of Medicine Saint Louis, MO United States; 3 Department of Health, Human, and Biomedical Sciences University of Houston-Clear Lake Houston, TX United States; 4 Center for Medical Ethics and Health Policy Baylor College of Medicine Houston, TX United States; 5 College of Nursing University of Central Florida Orlando, FL United States; 6 Neamah Health Consulting Boston, MA United States; 7 Clinical Strategy and Research Team Modern Health San Francisco, CA United States; 8 Department of Population Health Science Weill Cornell Medicine Cornell University New York, NY United States; 9 School of Medicine Yale University New Haven, CT United States; 10 Fred Hutchinson Cancer Center Seattle, WA United States; 11 Department of Biomedical Informatics University of Utah School of Medicine Salt Lake City, UT United States; 12 Psychology Department Old Dominion University Norfolk, VA United States; 13 Virginia Consortium Program in Clinical Psychology Norfolk, VA United States; 14 Department of Psychology Concordia University Montreal, QC Canada; 15 Department of Nutritional Sciences University of Texas at Austin Austin, TX United States; 16 Department of Population Health Dell Medical School University of Texas at Austin Austin, TX United States; 17 Department of Organizational Systems and Adult Health University of Maryland Baltimore, MD United States; 18 Division of Public Health Sciences Department of Surgery Washington University School of Medicine St. Louis, MO United States

**Keywords:** digital health, health equity, mobile health, mHealth, health care access, digital divide, behavioral medicine, implementation, mobile phone

## Abstract

The potential and threat of digital tools to achieve health equity has been highlighted for over a decade, but the success of achieving equitable access to health technologies remains challenging. Our paper addresses renewed concerns regarding equity in digital health access that were deepened during the COVID-19 pandemic. Our viewpoint is that (1) digital health tools have the potential to improve health equity if equitable access is achieved, and (2) improving access and equity in digital health can be strengthened by considering behavioral science–based strategies embedded in all phases of tool development. Using behavioral, equity, and access frameworks allowed for a unique and comprehensive exploration of current drivers of digital health inequities. This paper aims to present a compilation of strategies that can potentially have an actionable impact on digital health equity. Multilevel factors drive unequal access, so strategies require action from tool developers, individual delivery agents, organizations, and systems to effect change. Strategies were shaped with a behavioral medicine focus as the field has a unique role in improving digital health access; arguably, all digital tools require the user (individual, provider, and health system) to change behavior by engaging with the technology to generate impact. This paper presents a model that emphasizes using multilevel strategies across design, delivery, dissemination, and sustainment stages to advance digital health access and foster health equity.

## Introduction

Behavioral medicine plays a key role in testing and implementing newly developed digital health technologies and evaluating their use in care delivery, including recognizing unique opportunities and challenges to equitable health services [1,2]. Along with many other health-related specialties, behavioral medicine responded to the COVID-19 pandemic with the rapid deployment of digital health and faced concerns that digital health disparities deepened during the pandemic [3-6]. Digital health tools, which include mobile health, health information technology, wearable devices, telehealth, and telemedicine [7], were rapidly implemented in lieu of in-person activities to limit the spread of COVID-19 while trying to ensure that access to care was uninterrupted [8]. While benefits of digital health were seen in some population groups, the pandemic also exposed long-standing inequities in health and health care, especially access to care. Often described as the “digital divide” [9], the gap between those with access to digital technologies and those without [10,11] came into stark relief during the pandemic. Thus, this again highlights the need to prioritize digital health equity while sustaining the momentum of the paradigm shift in health care delivery to digital health technology [12,13].

Behavioral medicine brings unique perspectives to bridging the digital divide. Other health care fields join behavioral medicine in conceptualizing more comprehensive models to identify strategies that address barriers and enhance facilitators to ensure greater equity in digital health. A body of work within behavioral medicine has focused on improving access to care [14] and reducing health disparities [1]. Alcaraz et al [1] developed the ConNECT framework that links behavioral science with health equity and provides strategies, including harnessing technology, focused on marginalized subgroups to inform practice and policy to promote health equity. Harnessing technology can promote equity by, for example, bridging accessibility barriers; using seamless technological adaptations (eg, language, literacy, or cultural tailoring); or scaling treatment approaches. These considerations are critical given that digital technologies have been repeatedly shown to exacerbate health disparities if equity-promoting approaches are not considered at every stage of the design of digital tools [15].

Complementary models such as the Digital Health Equity Framework address multilevel dimensions of population access to health care and build from the socioecological model to demonstrate that multilevel factors such as digital literacy, community infrastructure, and policy must be addressed to mitigate inequities [4]. *Access* is described by Levesque et al [14] in health care access conceptual framework as an individual’s opportunity and ease of using appropriate services in proportion to their needs [14]. Although access is related to price, quality, and availability, it is also impacted by individuals’ behaviors; individuals must use the service, which depends on many factors, such as an individual’s preferences, values, knowledge, age, abilities, and level of clinical need.

Bringing complementary models together can identify unique opportunities for digital health access that are more vital in addressing health inequity [1]. Behavioral scientists have contributed to this growing field of digital health equity in areas such as integration of equity principles into digital interventions [16], inclusion of user-centered or co-design methods for cultural tailoring of digital health interventions [17], development of new ways to engage historically marginalized groups [18], and engagement of the community through formative evaluations when digitizing interventions for dissemination [19]. As the proliferation of digital health technologies for behavior change continues, the potential for wide-scale public impact across disciplines (eg, engineering, psychology, nursing, and public health) and disease and illness foci (eg, diabetes, cancer, obesity, HIV, and mental health) could be further realized if strategies to promote equitable access are used at all levels of the socioecological model. Our viewpoint is that (1) digital health tools have the potential to improve health equity if equitable access is achieved, and (2) improving access and equity in digital health can be strengthened by considering behavioral science–based strategies embedded in all phases of tool development. Our paper is the first to integrate equity (ConNECT) and access frameworks (Levesque et al [14]) to explore how dimensions of access impact digital health equity comprehensively. This paper aims to examine key drivers of inequities at multiple access levels and provides actionable strategies to promote digital health equity across stages of development, delivery, dissemination, and sustainment.

## Methods and Guiding Principles

Health equity is an important focus within behavioral medicine, specifically the Society of Behavioral Medicine [20]. This paper aims to articulate a behavioral medicine perspective to improve digital health equity. Coauthors represent a multidisciplinary group of behavioral scientists from the Society of Behavioral Medicine spanning academia, industry, and nonprofit organizations with expertise in informatics, health services research, dissemination and implementation science, and health disparities research. Leveraging our working group’s diverse experiences and expertise, we cataloged the key challenges of equitably implementing, disseminating, and sustaining digital health interventions among diverse populations and settings. As we compiled these challenges, six guiding principles emerged to generate a shared understanding critical to clarifying our perspectives that follow.

First, the field of digital health requires the application of overarching ethical principles and several unique considerations as outlined in the Ethics Checklist for Digital Health Research: engagement of end users; informed consent; equity, diversity, and access; privacy and partnerships; regulation and law; and return of results [21].

Second, beyond ethical considerations of technology, connectivity, monitoring, and data management, it is critical to consider social, cultural, economic, infrastructure, policy, and other intersectionalities to promote digital health equities.

Third, digital health tools can be designed for and disseminated directly to individuals (eg, wearable devices) or can be used or delivered by health care organizations and teams. These distribution pathways have unique implications for implementation, dissemination, and sustainability (eg, funding sources) that result in unique drivers of inequities at multiple socioecological levels (providers, individuals, health care systems, third-party payors, and public health).

Fourth, understanding the groups that are underserved by digital health is a critical step. This paper refers to these groups broadly as underserved populations. By this, we mean people and communities disadvantaged by our systems and policies, including racial and ethnic groups, non–US-born persons, people with lower incomes, rural communities, people with disabilities, lesbian, gay, bisexual, transgender, queer, intersex, and asexual communities, and people who are incarcerated. Our intention is to speak broadly and without stigma to advance digital health for all.

Fifth, behavior change is inherent to the success of digital health interventions. All digital tools require behavior change; in other words, they require the user (individual, provider, and health system) to use the technology to generate impact for positive health outcomes. Digital tools may also have behavior change (eg, blood pressure self-monitoring and promotion of physical activity) as their primary purpose. Sometimes, behavior change must occur at multiple levels to generate the intended outcome. For example, a clinical decision support tool to promote cancer screening requires the clinician to change the way in which they are delivering care and requires the patient to get screened.

Sixth, to achieve equity, multilevel strategies (individual-, developer-, organizational-, and policy-levels) are necessary to consider during the design, dissemination, implementation, and sustainment stages of digital health interventions.

## Theoretical Framework: Limited Access Drives Digital Health Inequity

**Figure 1:**
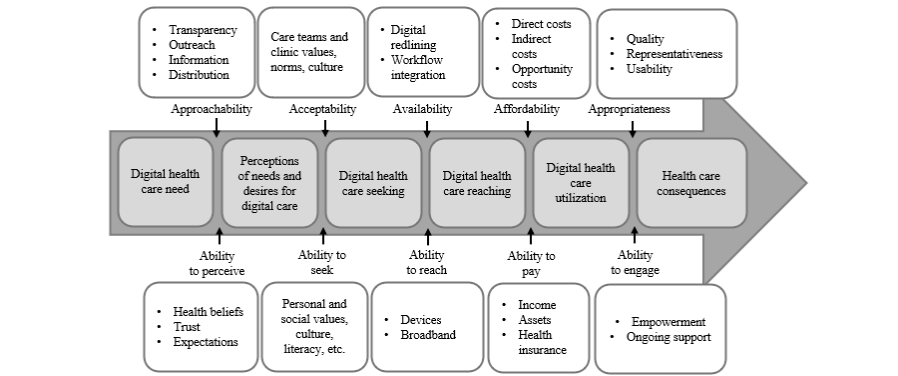
A conceptual framework of drivers of inequitable access to digital health. Adapted from the health care access framework by Levesque et al [14].

We propose that overlaying a behavioral medicine perspective with multilevel, intersecting strategies that address digital health access inequities can yield greater reach, adoption, and impact of digital health across diverse populations. Applying the access framework by Levesque et al [14] to the digital divide can promote equity by considering the multiple dimensions of access to digital health: approachability, acceptability, availability, affordability, and appropriateness. Our adaptation of this framework specifies a digital access application, rather than health care access more broadly, and integrates intersecting factors influencing digital health accessibility and accounting for the barriers experienced at multiple steps. We found the framework by Levesque et al [14] useful for examining specific digital health inequities and presenting our application within the 5 access dimensions (Figure 1). We adapted specific digital health access barriers, focusing on multilevel factors that may generate differences in one’s desire or perception of need and the ability to seek, reach, pay for, and engage with digital health [14,22]. Our framework uses a socioecological perspective to describe multilevel drivers of inequities and strategies to generate equitable access. This is critical, as digital health tools may be used by individuals and health care teams and disseminated through companies and organizations, as noted in principle 3 in the Methods and Guiding Principles section.

## Drivers of Digital Health Inequities Using the Health Care Access Conceptual Framework

### Approachability: As a Driver of Digital Health Inequities

A digital health tool is approachable if an individual is aware of the tool and perceives a need for it. However, awareness of digital health tools may vary among social and geographical population groups. Levesque et al [14] propose that transparency of data collection or use, tool content, and outreach activities all contribute to the approachability of health care services. However, outreach activities are often not tailored to diverse subpopulations within the intended audience and, therefore, do not equally increase awareness and produce the intended outcome for all groups, especially underserved populations [23,24]. Additionally, Levesque et al [14] indicate that individuals must perceive a need for care (ie, the digital health intervention), which is impacted by factors such as health knowledge and health-related beliefs. Individuals may lack a perceived need for a digital health tool that has not established credibility, particularly among underserved populations, and faces fundamental challenges with efficacy, validity, and compliance.

### Acceptability: As a Driver of Digital Health Access Inequities

The acceptability of a digital health tool influences the decision to initially adopt and continuously engage with the digital tool, which is a precursor to receiving the intended health benefit [1]. Acceptability is interconnected with the approachability of a digital health tool by influencing one’s perceptions of needing the tool as well as the willingness to share about the tool with others and influences one’s perceptions of the appropriateness. Differences in culture, values, digital health literacy, numeracy, physical ability, visual acuity, hearing, and structural impediments influence an individual’s acceptance and interactions with digital health tools [22]. Digital health tools that do not account for unique social and cultural factors, particularly among historically underserved groups, are often less relatable [25] and increase people’s hesitance to use and benefit from them. This discrepancy in the acceptance of the digital health tool may be due to the failure of digital developers to incorporate the diverse views of end user perspectives, including the individual user and those who may deliver (eg, health care teams) the tool, in the conceptualization and design phases [26-28]. This results in tools that are unintentionally designed with features that are unappealing or inaccessible for the individuals and communities for which they are intended [29].

People who are visually impaired, color blind, older adults, and people from different races, ethnicities, sexual orientations, or abilities all have unique needs and preferences that should be considered [26]. Beliefs about the efficacy of the digital tool may influence initial acceptance and continued use [30]. For example, beliefs regarding the provider’s ability to deliver quality care using telehealth were associated with fewer telehealth visits during the COVID-19 pandemic among African American individuals with diabetes [31], and overall, African American individuals have limited trust in the health benefits of digital health interventions [32]. Concerns with data privacy and security also reduce trust or willingness to use digital tools, especially among African American individuals [33].

A critical consideration is whether the care team also believes in the ability and security of the tools to improve care and patient outcomes, specifically for underserved populations [34]. The care team may be aware and hold beliefs about digital health tools that reduce their acceptability and impact the health care team members’ and organizations’ readiness and willingness to use a digital tool or recommend one to the patient. If a care team member does not share about a digital health tool, it may reduce a patient’s awareness (approachability) of the tool. In addition, the continued lack of resources and support for use (eg, IT support and training) increases perceptions that digital tools disrupt workflows and delivery of care [35,36]. These fundamental challenges may compromise credibility among health care providers and executives and further impact the approachability and integration of digital health into the health care system.

### Availability: As a Driver of Digital Health Inequities

Availability is the ability to reach and use digital health tools in a timely manner. Availability is largely dictated by an individual’s access to devices (eg, smartphones and computers) and broadband internet. In 2021, 85% of Americans owned a smartphone, and 77% had broadband access, making smartphones and SMS text messaging intervention an appealing approach for widespread reach [37,38]. Access to broadband is so critical to health that it has been advocated for as a social determinant of health [39]. Although broadband access continues to rise, limited access has been associated with specific racial minority groups, lower education, lower income, living in rural areas, and the process of digital redlining (ie, the creation and perpetuation of inequities between marginalized groups through technology) [37,40]. Access to broadband internet also impacts how health interventions are delivered. For example, individuals who lack access to the internet at minimum connection speeds will typically be unable to access digital interventions that depend on features such as streaming and video calls (eg, Zoom).

At the organization and systems levels, enhanced access to services that support digital health is a concern. Health care facilities in underserved rural and urban communities have limited resources for implementing advanced digital functions, such as telehealth services [41]. This has increased disparities in access to mental health services among children in low-resourced areas where they are unable to access internet-based visits with providers [42]. Availability within a clinic or organization may also be limited by poor design and implementation planning, which leads to failure to integrate the digital health tool into the workflow and inadequate training health care teams to deliver digital health interventions routinely [43]. Lack of infrastructure and resources (eg, staff, IT support, internet access, and devices) within clinics has been cited as a continued barrier to using digital health tools despite their rapid growth [35,36]. Furthermore, academic researchers often rely on time-limited grants to develop digital health tools, and these grants provide limited financing for the long-term operation, hindering the scalability and availability of digital health tools beyond the pilot stage of the intervention.

### Affordability: As a Driver of Digital Health Access Inequities

Affordability is the economic capacity for people to spend resources and time to use appropriate services [14]. In digital health, this may be costs for the individual user (eg, patient), staff delivering care, and organization providing the digital health intervention (eg, clinic and hospital system). Costs may include obtaining digital devices or applications (eg, physical activity trackers, blood pressure monitors, or smartphones, and apps); connectivity for internet data transfer; and geolocation, which may be particularly prohibitive for individuals or organizations in underserved communities [29,44-46].

At the clinic level, disparities exist in the type of informatics systems afforded (eg, electronic health record [EHR] systems) and if and how new technologies can be easily adapted. For example, the digital infrastructure in community clinics may be less adaptable than the systems used by academic medical centers (eg, lack of skip logic, display logic in data collection forms, and lack of ability to place a referral from a questionnaire in the EHR). These system design challenges hamper health care by diminishing the process of collecting information, in particular, on patient social and behavioral determinants of health and making referral to appropriate services. Inequities may also stem from disparate costs during the development of digital health tools, as the necessary inclusion of end users in the design process of digital health solutions is time-intensive and costly [47]. In addition, consideration of the costs of maintaining digital health tools, the necessary digital infrastructure, and technical support is necessary for equitable, effective, and efficient digital care.

The unique approach to affordability by Levesque et al [14] provides insight into costs not only as a mechanism of payment for a service or tool but also as indirect costs (eg, time to use the tool) and opportunity costs (eg, time needed to learn how to use a new digital health tool). In particular, populations with low digital literacy (eg, older generations) may require more time and support to learn how to use digital tools, and this increases the opportunity costs and limits their ability to access digital health tools at the same rate as other users [48,49].

### Appropriateness: As a Driver of Digital Health Access Inequities

Appropriateness refers to the ability of digital health tools to adequately address an individual’s needs. When a tool is appropriate, individuals remain engaged and experience the benefits. Personal factors (eg, digital health literacy, language, stigma, and age) and technological factors (eg, not adapted for different abilities, confusing interface, and lack of structure) drive inequities in engagement and effectiveness that impact the ability of a digital health tool to meet the needs of the user. The rapid digitization of health care has implications for health care providers who are serving these diverse populations, including older adults who are more likely to have low digital literacy and may not be as able to benefit from these technologies [3]. Furthermore, low levels of digital literacy among older adults increases loneliness and reduces health seeking behaviors and quality of life [50,51]. As an indicator of appropriateness, a systematic review on the usability of digital health interventions for anxiety, depression, and somatoform disorders found that participants’ initial beliefs and the amount of support and personalization that they received influenced engagement and dropout [52].

Appropriateness can also be conceptualized at a population level by evaluating who is accessing and benefiting and who may not be benefiting [32]. Digital health studies often lack both the measurement of social and digital determinants of health and the representation of people from underserved communities that would enable research evaluations on appropriateness and equitable effectiveness [20]. By being excluded from intervention efficacy and effectiveness studies [53-55], underserved populations experience health data poverty [56] or the inability for some individuals or groups to benefit from innovation due to lack of representation. In addition, data-driven interventions (eg, machine learning techniques) built from homogenous samples risk being ineffective or harmful to groups not included in the initial design process and could increase the digital divide [57]. Without understanding who is accessing and benefiting, developers cannot design digital tools to improve health and well-being for everyone and risk creating biased or racist technology [58]. Further exacerbating these issues are the systems-level issues such as racial disparities in research funding for behavioral medicine [59] and a lack of scholar representation from underserved populations [60,61] that impact the equitability of research evaluation and ultimately impact the appropriateness of digital health interventions. 

## Proposed Strategies to Improve Equitable Access

### Overview

**Figure 2:**
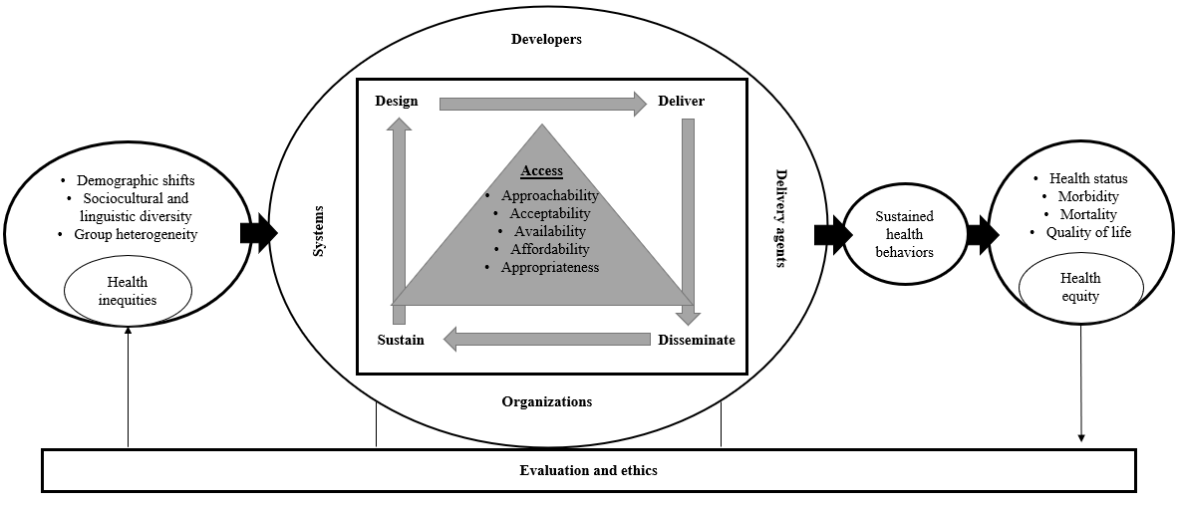
A model for advancing digital health access to foster health equity adapted from the ConNECT framework and the health care access conceptual framework by Levesque et al [14]. The model considers health inequities in access, digital health implementation stages and actors, and the subsequent impact on sustained health behaviors and health outcomes and equity.

To address these drivers of inequitable access, we propose a strategic model (Figure 2) for advancing digital health access to foster health equity. The digital health model, adapted from the ConNECT framework [1] and health care access conceptual framework by Levesque et al [14], considers health inequities in access (far left), implementation stages and actors (middle), and subsequent influences on health behaviors and more equitable health outcomes (far right). Strategies to improve access are presented and summarized in Multimedia Appendix 1 [26,27,29,35,49,56,62-85]. These include design strategies followed by delivery, dissemination, and sustainability strategies to address digital health access inequities. Consideration of subsequent phases of delivery, dissemination, and sustainability during the design phase (ie, designing with the end in mind) can support sustained impact on health behaviors and health equity [86]. Considerations of evaluation and ethics should be applied across all stages to further ensure equitable access. While many levels of influence exist, we focus primarily on the developers, delivery agents, and systems as key actors for proposed strategies. We consider developers to include academics, industry, and governmental or nongovernmental organizations that develop digital health solutions in collaboration with end users. Delivery agents include individuals who provide or deliver the solution to the end user (eg, clinicians and health care team members) and their organizations (eg, clinics, hospitals, broadband suppliers, and other organizations). Systems include the policies and funding structures in which users and suppliers are embedded.

### Design Strategies

Digital health tools designed using a theoretical basis, using behavior change techniques and in response to an end user’s needs and desires (eg, health issues perceived as critical) and background (eg, social identities, digital and health literacy, language, and stigma) can improve their acceptability, approachability, and appropriateness [87]. Developers improve access by determining and enlisting the input of their customers and target users, whether the tool serves a broad population or offers more personalized support to people with specific social identities (eg, health apps for different races, ethnic, or age groups) [88]. Design approaches that strive for a one-size-fits-all end product may not address the unique challenges faced by different populations, ultimately reducing use and impact of the digital tool.

Customer discovery and value proposition design, a form of stakeholder engagement based on marketing and LeanStartup business methods [89], can be used to understand the problem and articulate the product’s hypothesized unique value proposition relative to alternative options available to end users. This approach aims to identify a need or clinical problem requiring a solution, articulate a strong value proposition, and identify outcome measures that are meaningful to users to demonstrate efficacy and achieve buy-in [63,65,90]. Customer discovery has been applied to sustaining health informatics innovation and is foundational to the Innovation-Corps program for academic entrepreneurs developed by the National Science Foundation and adapted for health researchers by the National Center for Advancing Translational Sciences [63,91].

User-centered or participatory design methods may promote equitable access by designing tools that are relevant for different lived experiences and provide the information that individuals want, delivered how they like to receive it, and share it in a way that is secure and appeals to them in a user interface, resulting in a tool that is useful and accessible [92-95]. However, this approach is only successful when the intended users are broadly represented and engaged in the design process, underlying data, and product evaluation. Ensuring the intended audience is well-represented within design sessions and communicating who the tool was designed for can be challenging due to the intersectionality and diversity of individuals. Remaining cognizant of historical drivers of individual- and population-level inequities, including digital and health literacy and numeracy [48]; linguistic barriers [49]; and the uptake of a digital intervention for individuals with physical or cognitive limitations, including those more common among older adults age (eg, visual impairment), throughout design (and subsequent phases of implementation) is critical [96].

Equity of digital health tools is improved by undergoing usability testing by diverse, underserved individuals, including those of different generations, leveraging user experience methods, such as heuristic evaluations (does the tool meet the 10 general principles for user experience design and provide a usable experience?) and unmoderated tests (what is working and not working about the experience?). User testing may reveal technological aspects that can improve engagement from underserved groups, such as a simple interface, succinct content, reminders, feedback on progress, and acknowledgment of achievements [97]. The expansion of funding models to adequately support the described iterative design processes is critical to developing a product with the potential to be effective and equitable for underserved populations.

### Delivery Strategies

Intentional implementation and sustainability plans codeveloped with the end users are critical to the integration of these solutions into routine practice to make them an integral, consistent part of patients’ experience in health care. Ross et al [35] provide a nice example of using a systematic approach to implementation planning and execution. We urge others to share their implementation plan, including specific strategies, the plan development process, and adaptations made during delivery to generate a shared learning of the best approaches to implementing digital health solutions. The Expert Recommendations for Implementing Change provides discrete implementation strategies that are widely applicable and may be specified for digital health implementation in clinical care [98-100].

Ethnography may be used to observe and generate workflows that clarify the “patient journey” and points in the care visit where and by whom digital health tools should be used [68]. In addition to workflows or process maps, implementation plans may include strategies to address device ownership, broadband access, and digital health literacy screening as part of routine practice [101]. Understanding the ability of individuals to use a digital tool and the number and types of devices available in a household and whether those devices have sufficient bandwidth to support access such as telehealth conferencing is critical. Continued reimbursement for telehealth visits is necessary for successful implementation [102]. Offering digital health interventions in various formats (eg, virtual, real-time web-based help, in-person, and home assistance programs) may improve the ability to engage diverse, underserved populations [102].

Adequate training and ongoing technical support are needed for the end user to engage and benefit from the digital health tool successfully. Some clinics use a community-based navigator who could be trained to explain digital solutions to patients; connect patients to low-cost desktop, tablet, and mobile equipment and broadband or mobile data via local partnerships and federal subsidies; and provide ongoing support. At a systems-level, health care organizations should partner with community colleges, public libraries, and other community-based organizations to develop education and skill-building programs to address digital literacy gaps among underserved populations. Capacity (eg, staffing and time) of health care and community-based organizations and funding may be a challenge to institute and sustain these types of partnerships.

### Dissemination Strategies

Active and tailored dissemination of digital health tools to the target audience through determined channels and planned strategies will increase awareness (ie, approachability) and access to digital health solutions [103]. Communicating about digital health using audience segmentation, that is, the process of dividing your audience into segments and matching your communication channels and strategies to meet their preferences and needs, is necessary to increase awareness among historically underserved populations (eg, African American individuals and people living in rural communities) [104].

Forging a partnership between academics and industry is a unique strategy for academics to outsource the commercialization mindset and use strategies to improve widespread access. Using a flexible business and revenue model beyond the organization claiming intellectual property rights may reach more organizations and individuals [105]. One dissemination strategy may be to develop a platform, such as the one developed in Germany [76], where evidence-based digital health tools can be easily found. Criteria for defining what classifies as evidence-based, generating buy-in from health care providers, and maintaining such platform for universal use may present challenges to implementing this strategy [76]. Another crucial strategy is to support the early adopters of these innovations and to make their use of and benefit from the digital health tool observable. Strategies of crowdsourcing innovators and citizen science platforms support the sharing of tools that seek to solve scientific challenges that are important to the user [106-109]. Furthermore, involving health care workers from underserved populations as trusted messengers of digital health solutions may also increase reach and adoption among diverse groups [110]. Finally, and perhaps most importantly, it is necessary to create and foster an organizational culture of tolerance and patience for change and adoption for new innovations within health care institutions because diffusing innovations from early adopters to other segments in society requires significant time and energy from all stakeholders [111,112].

### Sustainability Strategies

Addressing disparities in affordability is particularly critical to sustaining access to digital health solutions. When scaling up an effective digital health solution, a sustainable funding plan must be developed from the outset to support long-term growth, which may require private-public partnerships. Government subsidies for broadband subscriptions and data charges for mobile health applications may help reduce individual costs. Additionally, broadband access can be expanded through community infrastructure, such as broadband hot spots in public spaces, including libraries and community centers [102]. McCall et al [40] recently outlined multilevel strategies to combat digital redlining (eg, intentional lack of investment in broadband) and expand broadband access and quality to all communities [113,114].

At the policy-level, regulations are needed that ensure internet service providers build infrastructure that meets the minimum requirements for high-speed internet, as defined by the Federal Communications Commission [40]. Renewed funding of government subsidies and programs such as the Regional Health Information Technology Extension Centers, which successfully promoted the adoption of EHRs in community clinics nationwide up until 2015, should be renewed to support clinics in underserved communities [77,115,116]. As the COVID-19 pandemic has highlighted, offering telehealth services can increase access to care and support underserved populations. Continued reimbursement for telehealth visits by both state Medicaid programs and the Centers for Medicare and Medicaid Services is critical to sustain improved access.

### Overarching Considerations: Evaluation and Ethics

Evaluating effectiveness of digital health interventions and sharing the results among underserved target populations is critical to improving approachability of digital health tools among these groups. Process evaluation that includes examination of metrics, such as acceptability, is critical throughout design, implementation, dissemination, and sustainment phases. Examination of acceptability, for example, may provide insights into reasons for low use or effectiveness and implications for the fidelity of both delivery and receipt of the intervention [117]. Evaluation ensures solutions are inclusive, equitable, and appropriate to address a health problem and to solve critical gaps in health care delivery. Implementation science and community-based participatory research literature can provide established approaches to consider in evaluation of digital health tools [118-120]. If digital health equity is to be realized, developers need a strong research focus with expanded funding mechanisms and support for implementing and evaluating innovative designs and analyses (eg, stepped-wedge designs, sequential multiple assignment randomized trial designs, and pragmatic randomized controlled trials) that simultaneously consider effectiveness and implementation (eg, engagement and integration in workflow) [43]. Digital health companies are growing rapidly and should include research teams to use these methodologies to validate the effectiveness of these technologies on health and health equity outcomes. The need for collaboration by researchers and digital health companies has been acknowledged through funding mechanisms, such as the National Institutes for Health (NIH) Small Business Innovation Research and Small Business Technology Transfer, that support research or business collaboration. These NIH funding mechanisms provide great opportunities for digital health solutions created in the private sector to benefit from the research expertise of clinical scientists and evaluate their solutions early in the process.

When considering the appropriateness of digital health tools for achieving the intended impacts across different demographic groups, we must also reduce health data poverty [56] by being advocates for populations who lack data, incorporating deliberative research and citizens’ juries (ie, inclusion of the community in decision-making), and communicating with people on how their data are being used and protected. It is critically important to create data sets that are representative of marginalized and underrepresented populations by using community-based participatory research methods and encouraging data sharing initiatives across digital health tools [20,56]. Justifying exclusion criteria should be provided to ensure that we are engaging diverse participants in digital behavioral health interventions and evaluation [46]. In addition, to address structural issues, funders, regulators, and policy makers should require digital health interventions and tools to perform appropriately and be usable for different populations (especially historically underrepresented groups) and settings [56]. In addition, and to act on multiple levers of change within the society, funders may also create separate funding mechanisms to specifically support the development or adaptation of digital health tools tailored for historically marginalized groups. In case successful digital health tools were already created for the mainstream population, these mechanisms can ensure that separate digital health tools with similar missions are available for marginalized groups with specific needs.

All tools should use ethical principles and theories in design, including confidentiality, inclusivity, and transparency [84]. To increase trust in digital health tools, there needs to be greater transparency in transferring and sharing data collected using behavioral digital technology [44] while maintaining privacy and security and returning results to patients or participants [27,29,84]. Core elements of privacy to ensure when using personal and remote individual digital monitoring include safety and security in digital services for data collection, transfer, storage, management, and sharing [21,62,84,121]. The Connected and Open Research Ethics (CORE) tool may support researchers and institutional review boards with data privacy, transfer, and transparency to support digital health research [122]. CORE may be used to find or share informational resources (eg, institutional review board and consent documents) or receive feedback from experts. Using approaches, such as CORE, to improve confidentiality and transparency are particularly important when working with historically marginalized populations who have faced ostracization, discrimination, and even physical danger from privacy violations. For example, during the HIV and AIDs epidemic, lesbian, gay, bisexual, transgender, and queer individuals were fearful that their HIV and AIDs status would be disclosed and that they would face stigma at work or risk losing their job. More recently, individuals who menstruate have expressed concerns regarding data privacy and security when using period-tracking apps and the potential harmful consequences of these data being misused in a post–Roe v. Wade era. Outside health care, data privacy has implications for discriminatory advertising, racially biased policing, and the outing or surveillance of historically marginalized populations. Intellectual property, policies, and data governance structures must be addressed to safeguard personal behavioral data and rights that adhere to policy and law, especially for historically marginalized populations [44,84].

## Discussion: Focusing Future Solutions for Equitable Digital Health Access

### Overview

The “promises and perils” of digital tools to achieve health equity have been highlighted for over a decade [121,123], but success in achieving equitable access to health technologies remains challenging. Our paper addresses renewed concerns regarding equity in digital health access that were deepened during the COVID-19 pandemic [3-6]. Using behavioral, equity, and access frameworks allows for a unique and comprehensive exploration of current drivers of digital health inequities. It allowed a compilation of strategies that have the potential for actionable impact on digital health equity.

We propose a model that emphasizes using multilevel strategies across stages of design, delivery, dissemination, and sustainment to advance digital health access and foster health equity. Strategies were shaped with a behavioral medicine focus, as the field has a unique role in improving digital health access; arguably, all digital tools require the user (individual, provider, and health system) to change behavior by engaging with the technology to generate impact. Furthermore, digital health tools targeting health behaviors (eg, physical activity and mental health apps) have recently exploded [2]. Finally, a breadth of behavioral research offers critical evidence to draw upon from individual to population effects, providing a myriad of behavior change tools and strategies that have been shown to be effective, to have broad reach, and to have the potential to be widely disseminated and sustained.

Multilevel factors drive unequal access, so strategies require action from tool developers, individual delivery agents, organizations, and systems to effect change. We outline key strategies at multiple stages, albeit not exhaustive, to promote digital health equity to achieve the intended health outcomes and consequences (Multimedia Appendix 1). Multilevel solutions are highlighted for key producers of digital approaches and recommendations to engage diverse end users to develop representative, acceptable, and appropriate tools for the intended audiences. Once designed, digital health tools demonstrating usability and accessibility require further evaluation to address dissemination and implementation challenges at organizational and systems levels [20]. Successful and sustainable digital health approaches are supported by multifaceted strategies, culturally and linguistically appropriate methods, tailoring, establishment of mutual partnerships, and community engagement.

Implicit in our model is the potential for multidisciplinary and team-based approaches in applying recommended strategies. Given the important role of industry in creating new digital tools, exploring new models of academic, community, and industry partnerships is recommended to work upstream in addressing improved access [124-126]. While academics and industry approach development and evaluation differently [2], synergy could be found in blending their strengths of evaluation and innovation. Academics focus on design, intervention, and efficacy testing, infusing behavioral theory and evidence before disseminating to end users for input. They often start with a targeted product that is personalized to a specific population or social identity and, even more often, is constrained by funding that requires this sequence of development. Conversely, industry develops a product for a broad audience and immediately disseminates it, iterating the functionality with user feedback and eventually examining effectiveness (if at all). The resulting products may be either effective but not widely used or engaging but not meaningfully effective to result in positive health outcomes. While the goal should be effectiveness, there are opportunities to design digital tools to improve the health and well-being for both everyone and for specific groups. This mixed approach is critical as different health providers and systems serve different populations, and the goal of health equity is to meet the specific needs of their population. While a combination of tools for both broad and specific populations is likely needed to achieve health equity, using ethical principles of transparency to state for whom the tool is designed and is proven to benefit and perhaps, more importantly, for whom the tool may not benefit is important to ensure adoption, use, and dissemination to the right individuals to achieve positive health impacts.

Team science practices and experience can shape new ideas about forming digital health partnerships [2]. To begin, academics could consider hiring experienced commercial developers and designers on their team to infuse usability principles and integrate efforts toward sustainability from the outset of product development, and industry companies could consult with academic scientists or build an in-house team of experienced clinical researchers to integrate theory-based interventions and evidence-based practice. Behavioral science expertise could inform considerations of several issues, such as transparency and fairness in application software; how big data and self-monitoring may regulate or constrain behavior; how to obtain user consent for data use, especially by employers or insurance companies; and comparisons of in-person to digitally mediated health care [84]. Developing meaningful collaborations with scholars from diverse underserved communities who are health equity experts is critical in addition to initiatives to transform institutional culture through programs such as NIH Build and the National Research Mentoring Network [127]. Additionally, partnerships with community colleges, health departments, public libraries, and other community-based organizations are necessary for a bidirectional flow of information and resources that may support common goals of digital health access across systems, individuals, and national or state policies [128]. These relationships can bridge multiple levels [129] and consider and account for the interconnectedness and bidirectional nature of movement within and across internal (clinic or organization level) and external factors that shape digital health access.

### Limitations

While using behavioral, equity, and access frameworks allowed for a unique and comprehensive exploration of current drivers of digital health inequities and a compilation of strategies, a systematic literature search was not conducted. While the intent was to provide an overview of barriers, not using systematic methods may have limited our ability to generate content that is entirely comprehensive. This paper used a narrative search driven by the discussion and comparison of frameworks and drawn from the expertise of our multidisciplinary team. The authors are mainly behavioral scientists, and some are clinical providers; while they were culturally diverse, they may not reflect all perspectives across all demographic and cultural groups. This may limit or bias the perspectives of our report. To reduce biases, we were intentional about drawing from the literature and do acknowledge that this is a viewpoint paper that reflects some positionality of the authors.

### Conclusions

Health equity is an ethical imperative that digital health equity can contribute to achieving. Digital health tools have the potential to reduce health inequities when equitable access is achieved. Applying behavioral science perspectives to better understand the multilevel factors driving unequal access can contribute to solving existing and emerging health disparities. Addressing concerns that threaten the widespread benefit from effective and validated digital health tools to society and further prevent benefit for historically marginalized communities needs urgent attention to bring these solutions to those most in need. Transdisciplinary teams that use this integrated digital health equity model (Figure 2) and strategies across design, delivery, dissemination, and sustainability stages may significantly improve digital health access and health equity.

## Appendix

Multimedia Appendix 1 Strategies for developers, delivery agents, and systems to improve access and promote digital health equity.

## Abbreviations

CORE: Connected and Open Research Ethics
EHR: electronic health record
NIH: National Institutes for Health
