# Supplementary material for: Expanding a Behavioral View on Digital Health Access: Drivers and Strategies to Promote Equity
Source: J Med Internet Res. 2024 Aug 1;26:e51355. doi: 10.2196/51355 (PMC11327633; doi:10.2196/51355)
Supplement: Multimedia Appendix 1 [file jmir_v26i1e51355_app1.docx]

**Table S1.** Strategies for developers, delivery agents, and systems to improve access and promote digital health equity.

| Stage and strategy | Example | Actor | | | Impact on access |
| --- | --- | --- | --- | --- | --- |
|  |  | Developers | Delivery agents | Systems |  |
|  | | | | | |
| **Design** | | | | | |
| Use user-centered and participatory design approaches that include all people that represent your end user, being cognizant of including underserved individuals | - During development, draft web-based app content was shared with 2 focus groups of intended end users and a Community Advisory Board participated in the analyses of focus group data [27] - Formative evaluation using mixed methods informed the development of an mHealth^b^ intervention for improving patient care [62] | ✔ | —^a^ | — | Approachability, acceptability, and appropriateness |
| Design tools cognizant of literacy and numeracy, linguistic barriers, and physical and cognitive limitations | - Rodriquez et al [49] offer recommendations for ensuring patient portals address the needs of patients with limited English proficiency | ✔ | — | — | Approachability, acceptability, and appropriateness |
| Account for context in design to improve integration in workflows and systems | - A commercialization-readiness framework was applied to promote the sustainability of an electronic health data network beyond funding [63] - Workflow analysis was used for the design of an electronic health record–based tobacco cessation intervention in community health centers [64] | ✔ | — | — | Approachability and availability |
| Build from evidence-based theory and techniques | - Self-determination theory principles were applied in the development of a health information technology tool and informed recommendations for how to administer the tool [65] - Self-determination theory to inform the development of The CARE intervention and to strengthen autonomous motivation and perceived competence [29] | ✔ | — | — | Acceptability and appropriateness |
| Expand funding models to support early iteration with user feedback | - Broader participation by institutes in NIH^c^ initiatives that support phased preparatory research (eg, R03 and R21) in line with review of best practices for intervention development [66] | — | — | ✔ | Approachability, acceptability, and appropriateness |
| Conduct proper usability testing | - In-depth interviews with end users to develop personas and use cases, iterative feedback, and engagement of key stakeholders informed the development of a digital health adviser [26] - Laboratory-based testing with standardized patient to assess provider use of patient-generated data in a clinical decision support tool to promote patient-provider communication about diabetes management [67] | ✔ | — | — | Acceptability and appropriateness |
| **Delivery** | | | | | |
| Develop intentional implementation and sustainability plans with end users | - Normalization process theory informed implementation strategies, and iterative introduction was used to implement a digital diabetes management intervention in routine practice [35] | ✔ | ✔ | — | Availability |
| Use ethnography to observe and map workflows and points of integration | - Although not specific to digital health, ethnography has been used to understand health care decisions, care seeking behavior, and nonadherence [68] | ✔ | ✔ | — | Approachability and availability |
| Routinely and consistently screen for digital literacy and availability of devices and broadband | - Digital literacy assessment can be integrated with community engagement using valid and reliable survey instruments appropriate to the population of interest [69,70] - Designed and framed in linguistic language that is understandable to the users [71] | — | ✔ | — | Appropriateness |
| Provide upfront training and ongoing technical assistance for users | - Technical support for tool navigation was provided for an intervention for older women with HIV [29] | ✔ | ✔ | — | Appropriateness |
| Use a community-based digital navigator in clinics and organizations | - Integrating and evaluating mHealth tools in a clinic addressing patient-community health worker-clinician communication for Hispanic adults at risk for diabetes [72] | — | ✔ | — | Availability and appropriateness |
| Provide community-based education and skill-building programs to improve digital literacy | - Conducted a 2-week intensive training and skill development in preparation for the implementation of an mHealth formative evaluation study [73] | — | ✔ | ✔ | Appropriateness |
| **Dissemination** | | | | | |
| Engage in active dissemination across populations using various modalities and audience segmentation | - COVID-19 emergency information dissemination in different modes to large non–English-speaking populations was crucial for uptake and adherence [74] | ✔ | ✔ | — | Approachability |
| Engage with industry to bring a commercialization mindset to increase reach | - Establishing AIPs^d^ to ensure digital health solutions are evidence based without losing sight of commercialization plans | ✔ | ✔ | — | Availability |
| Use a flexible business model to overcome intellectual property rights | - Barbieri and Andreoni provide an analysis of the intellectual property rights in the field of mHealth [75] | — | ✔ | ✔ | Availability and appropriateness |
| Develop a platform, where evidence-based digital health tools can be easily found and prescribed to patients | - In Germany, providers can prescribe approved digital health tools available in the national directory [76] | — | ✔ | ✔ | Approachability and acceptability |
| Engage health care providers from minority groups to deliver digital tools to boost trust | - MiSalud Health, a digital health start-up focusing on health equity for Latino community, recruits Latino physicians to use their platform and serve their clientele | — | ✔ | — | Approachability and acceptability |
| Create an organizational culture of tolerance and patience for change and adoption of innovations | - The Innovation and Digital Health Accelerator at Boston Children’s Hospital was established to promote an organizational culture of adoption for new digital solutions | — | ✔ | — | Approachability and availability |
| **Sustainment** | | | | | |
| Renew funding programs and government subsidies | - Initiatives by the US Federal Communications Commission such as the Rural Health Care Program and the Connect2HealthFCC Task Force help promote telehealth access among underserved communities [77] | — | — | ✔ | Availability and affordability |
| Enact reimbursement models for digital health interventions, including telehealth | - In the United States, digital therapeutics are regulated by the FDA^e^ and labeled as class II medical devices. Private and public insurance (Medicaid and Medicare) provide coverage for digital therapeutics products. Examples include EndeavorRx by Akili Interactive, ReSET by Pear Therapeutics, and Bluestar by WellDoc [78] | — | — | ✔ | Affordability |
| Provide broadband at public locations (eg, libraries) | - To support broadband availability for all the American Library Association has published position statements and their advocacy efforts to achieve them | — | ✔ | ✔ | Availability |
| Implement policies to support broadband access for all | - To support broadband availability for all the American Library Association has published position statements and their advocacy efforts to achieve them | — | — | ✔ | Availability |
| **Evaluation and ethics** | | | | | |
| Evaluate effectiveness and share results among underserved target populations | - Used a participatory evaluation approach to evaluate electronic communications about COVID-19 risk among African American churches [79] | — | ✔ | — | Approachability |
| Use a team science approach to balance innovation with evaluation efforts | - NIH mHealth Training Institutes contribute to interdisciplinary team science approaches when designing mHealth apps [80] | ✔ | ✔ | — | Approachability, acceptability, and appropriateness |
| Regulate usability of tools across diverse populations | - Funders and policy makers could require or incentivize usability across populations and settings [56] | — | ✔ | ✔ | Acceptability and appropriateness |
| Ensure data sharing, privacy, and security are acceptable and transparent to users | - Data privacy concerns were explored in focus groups with older women with HIV and considered in app design and optimization [27] | — | ✔ | ✔ | Approachability |
| Use and fund innovative trial designs (eg, SMART^f^ trials) | - The MOST^g^ framework used to evaluate an intervention for African American emerging adults to improve asthma management [81] - SMART trial to assess if motivational tailoring improved diabetes management app engagement among people with diabetes in a primarily Hispanic community in New York City [82] | — | ✔ | ✔ | Appropriateness |
| Create data sets that are representative of marginalized and underrepresented groups | - Health Data Research United Kingdom gathers routinely collected National Health Service data through specific initiatives (eg, Health Data Research Alliance) [83] | — | ✔ | ✔ | Appropriateness |
| Apply and mandate ethical principles of confidentiality, inclusivity, and transparency | - Shaw and Donia [84] argue for the importance of a sociotechnical perspective of ethics in digital health that includes the broader sociotechnical system for ethical analysis | ✔ | ✔ | ✔ | Approachability, acceptability, and appropriateness |
| Foster meaningful collaborations with scholars of color who are health equity experts | - The APA^h^ Mental Health Technology Advisory Committee engages health equity experts with diverse perspectives to advance APA initiatives in the digital mental health space [85] | ✔ | ✔ | — | Approachability, acceptability, and appropriateness |

^a^Not applicable.

^b^mHealth: mobile health.

^c^NIH: National Institutes for Health.

^d^AIPs: Academic Industry Partnerships.

^e^FDA: Food and Drug Administration.

^f^SMART: sequential multiple assignment randomized trial.

^g^MOST: Multiphase Optimization Strategy.

^h^APA: American Psychological Association.
